# The prognostic value of tumor-infiltrating lymphocytes in colorectal cancer differs by anatomical subsite: a systematic review and meta-analysis

**DOI:** 10.1186/s12957-019-1621-9

**Published:** 2019-05-22

**Authors:** Yamei Zhao, Xiaoxu Ge, Jiawei He, Yi Cheng, Zhanhuai Wang, Jian Wang, Lifeng Sun

**Affiliations:** 1 Department of Surgical Oncology, The Second Affiliated Hospital, Zhejiang University School of Medicine, Hangzhou, Zhejiang Province People’s Republic of China; 2 Department of Cancer Institute, The Second Affiliated Hospital, Zhejiang University School of Medicine, Hangzhou, Zhejiang Province People’s Republic of China; 3 Departments of Oncology, The Second Affiliated Hospital, Zhejiang University School of Medicine, Hangzhou, Zhejiang Province People’s Republic of China

**Keywords:** Colon cancer, Rectal cancer, Subtypes of tumor-infiltrating lymphocytes, Prognosis, Meta-analysis

## Abstract

**Purpose:**

In colorectal cancer (CRC), whether the immune score can be used to predict the clinical prognosis of the patient has not been completely established. Besides, the prognostic values of tumor-infiltrating lymphocytes (TILs) in different anatomical locations, counting sites, and subtypes have been controversial. The purpose of this meta-analysis is to analyze and determine the prognostic value of TILs indices including TIL subsets, infiltrating sites, and anatomical sites.

**Methods:**

Relevant literature was obtained by searching PubMed and Google Scholar. The pooled hazard ratio (HR) of the overall survival (OS), disease-free survival (DFS), and cancer-specific survival (CSS) was computed to investigate the prognostic significance of CD3+, CD8+, CD45RO+, and FOXP3+ T cells.

**Results:**

A total of 22 studies involving 5108 patients were included in the meta-analysis. In CC, based on T cell subtypes analysis, the final results indicated that CD8+ and FOXP3+ infiltrating cells, but not CD3+ T cells were prognostic markers for DFS and OS. In addition, with regard to the counting location of TILs, subgroup analysis revealed that only high FOXP3+ infiltrates in the tumor stroma (ST) were significantly associated with OS (HR = 0.38, 95% confidence interval (CI) = 0.22–0.67, *P* = 0.0007), whereas in invasive margin (IM), high density of CD3+ infiltrating cells indicated increased DFS (HR = 0.76, 95% CI = 0.62–0.93, *P* = 0.008). At the tumor center (TC), high CD8+ T cells infiltration was associated with improved DFS (HR = 0.50, 95% CI = 0.38–0.65, *P* < 0.00001). In RC, whether CSS or OS, high-density TIL was associated with improved prognosis.

**Conclusion:**

In a single counting site, high-density TILs reflect favorable prognostic value in CC or RC. For CC, more prospective studies are needed to verify whether different anatomical sites affect the distribution of TILs and thus the prognosis of patients. For RC, further studies should analyze the prognostic value of the immune score.

**Electronic supplementary material:**

The online version of this article (10.1186/s12957-019-1621-9) contains supplementary material, which is available to authorized users.

## Introduction

Colorectal cancer (CRC) is one of the most common malignant tumors of the digestive tract globally. The latest report estimated that in all new cases of malignant tumors, the incidence and mortality of CRC accounted for 10.2% and 9.2%, respectively [1]. Presently, immunotherapy has become an important treatment for CRC [2, 3]. It has been shown that the tumor microenvironment (TME) determines tumor germination and progression, and the presence of TILs in TME plays an important role in the process of killing tumors by immunotherapy [4], mainly because the type 1 microenvironment with high tumor mutation burden (TMB) and inflammation gene signatures is more likely to elicit an effective immune response [5]. The interactions among various components of the immune microenvironment mediate the execution of an immune response, and this is closely related to a favorable prognosis in colorectal cancer [6–9]. Several immune cells contribute to an effective immune response, CD8+ T cells of TILs serve as cytotoxic effects, whereas CD4+ T helper cells (CD4+Th) promote clonal expansion of antigen-specific CD8+ T cells and production of IFN-*γ*, thereby promoting proliferation and functioning as the effector molecules of CD8+ T cells and NK cell [10, 11]. In contrast, the presence of Treg (CD4+CD25+FOXP3+T cells), a subset of CD4+ T cells, appears to cause tumor immunosuppression, especially in most solid tumors [12]. Thus, high FOXP3+Treg infiltration indicates unfavorable prognosis [13–18]. However, FOXP3+ T cells predict a favorable prognosis of colorectal cancer [19–21]. CD45RO+ T cell is a subset of memory T cell, whose gene expression patterns overlap with that of Th1 cells and cytotoxic T cells. High CD45RO+ T cell infiltration is closely related to favorable prognosis [9, 22].

In CRC, the predictive value of different subtypes of TILs varies with the infiltration site [23]. The latest immune score suggest that TILs can be used for immunological classification, as well as to predict prognosis of human tumor. Moreover, it is equivalent to or more efficient than conventional TNM staging (AJCC/UICC TNM classification) [24, 25]. Several scholars have suggested that CRC should be considered as a heterogeneous disease, and differences between proximal CRCs and distal CRCs not only manifest in epidemiology, tumor characteristics, but also in multiple clinical pathological factors, genetic and molecular characteristics [26, 27], as well the density of some immune cells, and the prognostic value of TILs [28, 29]. Fewer studies have combined the subtypes of TILs and the infiltrating sites with the anatomical sites of colorectal cancer to assess the association between each subset of TILs and the survival outcome. Therefore, this systematic review and meta-analysis were performed to explore the prognostic value of TILs and T cell subtypes in colon cancer or rectal cancer.

## Materials and methods

### Search strategy

We searched the PubMed for relevant studies up to November 2018 using the following search scheme: (colorectal neoplasms OR neoplasms, colorectal OR colorectal neoplasm OR neoplasm, colorectal OR colorectal tumors OR tumors colorectal OR colorectal tumor OR tumor colorectal OR colorectal carcinoma OR carcinoma colorectal OR colorectal carcinomas OR carcinomas colorectal OR colorectal cancer OR cancer colorectal OR colorectal cancers OR cancers colorectal) AND (lymphocytes, tumor infiltrating OR tumor-infiltrating lymphocytes OR lymphocyte, tumor-infiltrating OR tumor infiltrating lymphocytes OR tumor-infiltrating lymphocyte OR tumor-derived activated cells OR activated cell, tumor-derived OR activated cells, tumor-derived OR tumor derived activated cells OR tumor-derived activated cell) AND (prognosis OR risk OR recurrence OR mortality OR survival OR predict OR outcome OR significant OR impact OR detect OR relevant). In addition, Google Scholar and Clinical Trial databases were searched to retrieve additional studies and other reviews without any restrictions. The reference list of other meta-analyses was screened to identify additional studies. All included studies were limited to Homo sapiens as subjects and were published in English.

### Study exclusion and inclusion criteria

Two independent reviewers (YMZ and GXX) selected the retrieved studies based on the title and abstract. If the topic of a study could not be confirmed from its title or abstract, the full-text was evaluated. Any disagreements were resolved by discussing or negotiating with a third party (HJW). In this meta-analysis, studies that met the following criteria were included: (1) All patients in the original study underwent surgical resection of the primary lesion and were diagnosed by pathological examination; the subjects did not receive neoadjuvant chemoradiotherapy or immunotherapy. (2) Researches reported whether the site of infiltration of T lymphocytes was the in left-side or right-side colon or rectal. (3) Researches identified TILs or subsets of TILs (CD3+, CD8+, FOXP3+, CD45RO+) and reported their association with CSS, DFS, or OS. (4) Studies provided sufficient data to calculate hazard ratios (HRs) and 95% confidence intervals (CIs). (5) TILs or the subtypes of TILs were identified by HE staining, immunohistochemistry, or flow cytometry. Exclusion criteria include insufficient data or case reports, reviews, comment, letters, and conference abstracts. Noteworthy, we incorporated some references from ineligible articles which met the above inclusion criteria. In addition, if a study had multiple publications, the one with the most suitable data was selected.

### Data extraction

Four investigators (ZYM, XXG, CY, and JWH) independently selected articles and extracted data according to a prepared form. The following primary information was extracted: the name of first author; year of publication; the number of patients; primary survival endpoint (including CSS, DFS (RFS), and OS); T lymphocyte subtype; T lymphocyte counting site; cutoff definition; and use of multivariate or univariate analyses (Additional file 1: Table S1). Survival endpoints included HRs for OS, DFS (RFS), and CSS as well as the 95% CIs for the high group and low group of each T cell subtype at specific counting sites within tumors (TC, ST, or IM). HRs were acquired from multivariate or univariate analyses and estimated from Kaplan-Meier survival curves using previously described methods, if HR could not be obtained directly [30]. Any disagreements were resolved by discussing with a third participant (ZHW).

### Quality assessment and risk of bias assessment

The quality of each study was assessed by a pre-existing form derived from a study by Mei et al [31] and was first developed and applied by McShane et al [32] and Hayes et al [33]. The following factors were evaluated: (1) Did the study provide the inclusion and exclusion criteria? (2) Were the patients’ data prospectively collected? (3) Were the main prognostic patient and tumor characteristics presented? (4) Was the IHC or HE staining protocol specified? (5) Were staining evaluated by > 1 observer? (6) Was the study endpoint defined? (7) Was the time of follow-up specified? (8) Was loss during analysis or follow-up described? The score of each study on a scale from 0 to 8 is provided in the Additional file 2 Table S2.

### Definition of prognostic outcomes and statistical analysis

The OS was defined as the time from date of initial primary diagnosis of CRC to death due to any cause or end of research; the DFS was defined as the time from date of initial primary diagnosis of CRC until the time of disease recurrence or progression was firstly observed; and the CSS was defined as the time from the initial primary diagnosis of CRC to the last objective follow-up information or death caused by the disease. The Review manager software (version 5.3; Cochrane Collaboration, Oxford, United Kingdom) was used for statistical analysis and meta-analysis. Implement statistical analysis was used to evaluate the association between survival endpoint (OS, DFS (RFS), and CSS) and subtypes of TILs (CD3+, CD8+, FOXP3+, CD45RO+ T cell) in different anatomical regions (colon or rectal). Due to insufficient number of studies and data on partial T lymphocyte subtypes, subgroup analysis was only based on TILs located in colon cancer and then described the relationship between high density of CD3+, CD8+ T cells infiltration, and DFS, as well as high density of CD8+, FOXP3+ T cells infiltration, and OS.

The HRs and 95% CIs extracted from each study were used to assess the association between high-density TILs and survival rate. A pooled HR > 1 reflected undesirable survival in groups with high number of TIL subtypes. On the contrary, a pooled HR < 1 reflected a favorable survival rate. A *P* value < 0.05 was considered to be significantly different and was calculated by the *z* score and *t* test.

Heterogeneity among the studies was evaluated using Cochran’s chi-square-based *Q* test [34], with low, moderate, and high levels of heterogeneity corresponding to the *I*^*2*^ value of 25%, 50%, and 75%, respectively. A random effects model was used to calculate the total HR, when the *I*^*2*^ value > 50% or *P* < 0.05. Otherwise, a fixed effects model was applied. Sensitivity analysis was performed by changing the analytical model or excluding studies one by one while observing the stability of the results. The sources and reasons of heterogeneity were determined using a quality assessment form (Additional file 2: Table S2) reported by Mei et al [31]. Publication bias was determined by Funnel plots for colon and rectal groups.

## Results

### Literature search results

**Fig. 1:**
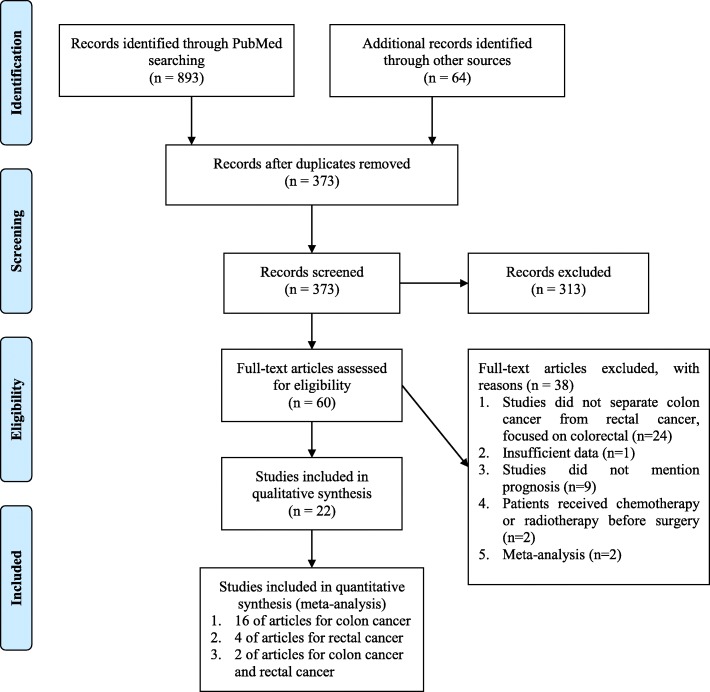
Flowchart of the study selection

A total of 22 studies were included in this meta-analysis. The clinical characteristics and search strategies are shown in Additional file 1: Table S1 and Fig. 1, respectively. Initially, 23 studies involving a total of 4731 patients were identified. Among them, 19 were colon cancer studies and six studies were related to rectal cancer. One study did not have sufficient data, and attempts to communicate with the author were not successful. Thus, the study was excluded. Ultimately, 22 studies were included in this study. All specimens were obtained from tumor tissues except for one study where it was taken from the blood [35]. Among the eligible studies, 16 research subjects had colon cancer [35–50], four had rectal cancer [51–54], and two had both colon cancer and rectal cancer [28, 55]. The counting sites for TILs in nine studies were ST, 10 studies were IM, and 17 studies were TC. HRs and 95% CIs were extracted from 21 studies, but HR and 95% CI were not available directly in one study. The survival data obtained from Kaplan-Meier curves was calculated using a spreadsheet as described previously [30, 43]. All pooled HRs, 95% CIs, and *I*^*2*^ test which were obtained by meta-analysis and subgroup analysis are summarized in Table 1. All studies had a quality assessment score of 4 or more, except for three articles, which scored 3 points (Additional file 2: Table S2).

**Table 1:** The pooled results and heterogeneity test (*I*^*2*^ test) of the meta-analysis in colon cancer

TIL subset	Tumor location	Outcome indicator	Counting site	No. of extractable data	Pooled HR	95% CI of HR	*I*^2^ test(%) of subgroup	*P* value of subgroup	Pooled *I*^2^ test (%)	*P* value	References (first author and year)
CD3+	Colon	CSS	IM, ST	2	0.58	0.46–0.74			0%	0.80	Richards 2014
		DFS	IM	2	0.76	0.62–0.93	39%	0.20	72%	0.003	Eriksen 2018, Flahevity 2016
			TC	3	0.59	0.15–2.28	84%	0.002			Guidobomi 2001, Lee 2010, Sinicrope 2009
			ST	1	0.14	0.02–1.07					Lee 2010
		OS	ST+TC	2	0.75	0.64–0.88			40%	0.14	Berntsson 2017
			ST	1							Lee 2010
			IM	1							Eriksen 2018
			TC	2							Guidobomi 2001, Lee 2010
CD8+		CSS	TC	1	0.55	0.39–0.79					Richards 2014
		DFS	IM	2	0.49	0.15–1.64	81%	0.02	72%	0.006	Eriksen 2018, Prall 2004
			TC	2	0.50	0.38–0.65	0%	0.36			Guidobomi 2001, Huang 2018
			TC+IM	1	0.65	0.50–0.85					Flahevity 2016
		OS	ST+TC	2	0.42	0.25–0.70	2%	0.31	64%	0.02	Berntsson 2017
			IM	2	0.31	0.04–2.69	79%	0.03			Eriksen 2018, Prall 2004
			TC	2	0.49	0.27–0.91	46%	0.18			Guidobomi 2001, Yoon 2012
FOXP3+		CSS	IM, TC	4	0.68	0.58–0.80			0%	0.79	Marlk 2017, Salama 2012, Ling 2014
		DFS	ST,TC	3	0.28	0.14–0.54			0%	0.57	Lee 2010, Correale 2010
		OS	ST	3	0.38	0.22–0.67	0%	0.54	44%	0.07	Lee 2010, Correale 2010, Yoon 2012
			TC	4	0.72	0.56–0.95	0%	0.41			Lee 2010, Xu 2013, Yoon 2012, Zeestraten 2014
			ST+TC	2	0.77	0.51–1.17	39%	0.20			Berntsson 2017
			Blood	1	3.78	0.92–15.52					Sellitto 2011
CD45RO+		DFS	ST, TC	2	0.22	0.10–0.53			0%	0.80	Lee 2010
		OS	TC, ST, NA	3	0.18	0.06–0.54			0%	0.46	Lee 2010, Lee 2013

*OS* overall survival, *DFS* disease-free survival, *CSS* cancer-specific survival; *TC* tumor center, *IM* invasive margin, *ST* tumor stroma; *TILs* tumor infiltrating lymphocytes, *FOXP3* Fork head box P3, *TC+ST* the immune score including tumor-infiltrating lymphocytes score of tumor center and tumor stroma, *TC+IM* the immune score including tumor center and invasive and invasive margin, *No* number

### Meta-analysis and subgroup analysis

#### The prognostic value of CD3+ T cell on the survival of colon cancer patients

**Fig. 2:**
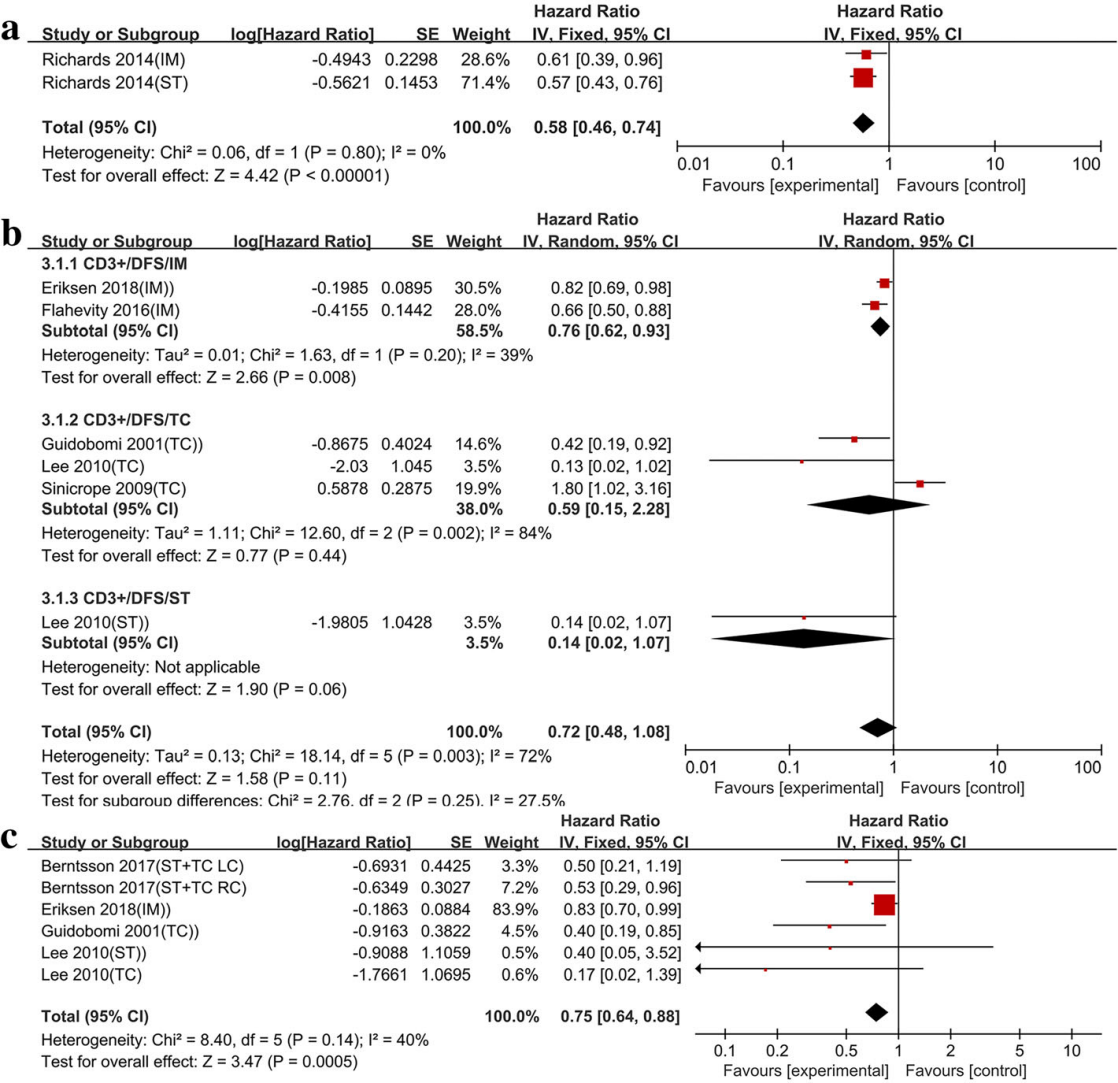
In colon cancer, forest plots of the fixed-effect or random-effect meta-analysis for the efficacy of CD3+ T cell for CSS (**a**), DFS (**b**) and OS (**c**), stratified analysis based on the location of lymphocyte infiltration, including the invasive tumor margin (IM), tumor center (TC), tumor stroma (ST). The horizontal bars indicate the 95% CIs. ST+TC: analysis of infiltration density of tumor infiltrating lymphocytes based on tumor stroma and tumor center; LC: left sided colon; RC: right sided colon

Data from six studies were pooled to evaluate the impact of CD3+ T cell on DFS and OS. However, only two studies explored the relationship between high CD3+ infiltrates and CSS. High CD3+ infiltrates correlated with improved CSS and OS unlike low CD3+ infiltrates (HR = 0.58, 95% CI = 0.46–0.74, *P* < 0.001; HR = 0.75, 95% CI = 0.64–0.88, *P* = 0.0005) (Fig. 2a, c). The pooled HR for DFS was 0.72 (0.48–1.08) indicating that the DFS of patients did not increase with CD3+ infiltration. Moreover, high heterogeneity was observed in DFS (*I*^*2*^ = 72%, *P* = 0.003) (Fig. 2b). In subgroup analysis, at the invasive merge, significant differences were observed between CD3+ infiltration and DFS (HR = 0.76, 95% CI = 0.62–0.93), but heterogeneity was significantly decreased (*I*^*2*^ = 39%, *P* = 0.20) (Fig. 2b).

#### The prognostic value of CD8+ T cell on the survival of colon cancer patients

**Fig. 3:**
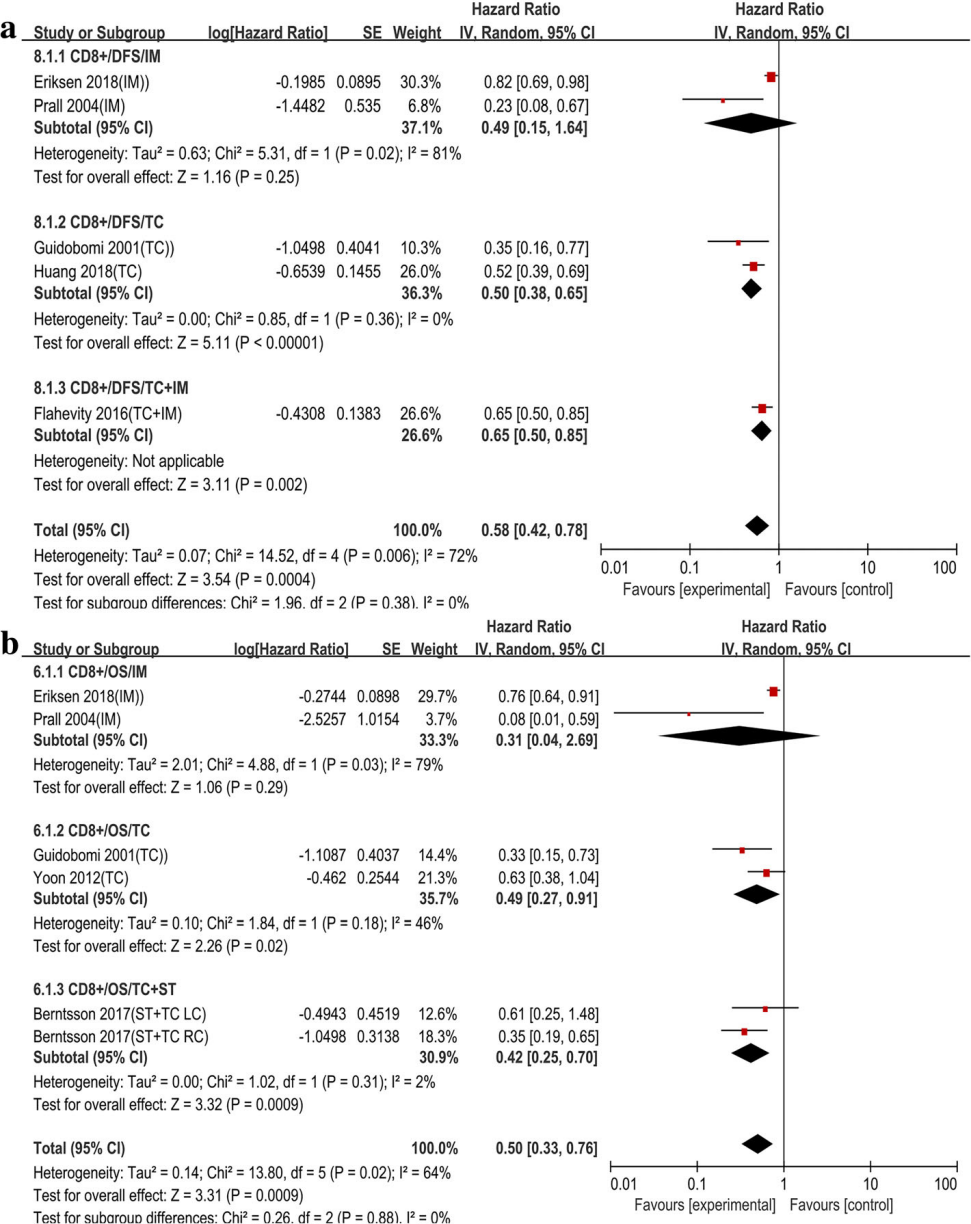
In colon cancer, forest plots of the random-effect subgroup meta-analysis for the efficacy of CD8+ T cell for DFS (**a**) and OS (**b**), stratified analysis based on the location of lymphocyte infiltration, including invasive tumor margin (IM), tumor center (TC), tumor stroma (ST). The horizontal bars indicate the 95% CIs. ST+TC: analysis of infiltration density of tumor infiltrating lymphocytes based on tumor stroma and tumor center; TC+IM: analysis of infiltration density of tumor infiltrating lymphocytes based on tumor stroma and invasive tumor margin; LC: left sided colon; RC: right sided colon

Five studies provided the HR and 95% CI for the correlation between CD8+ T cell and DFS, with the counting site of two studies located at the TC and two located at the IM. In the general analysis of the five studies, although the pooled HRs indicated an improved prognosis, high level of heterogeneity was observed (HR = 0.58, 95% CI = 0.42–0.78; *I*^*2*^ = 72%, *P* = 0.006) (Fig. 3a). The high heterogeneity was decreased in the subgroup analysis, especially in TC. However, the association between CD8+ T cells and DFS did not change (HR = 0.50, 95% CI = 0.38–0.65; *I*^*2*^ = 0%, *P* = 0.36) (Fig. 3a). Pooled analysis of studies concerning CD8+ in the IM did not indicate a prognostic impact regarding DFS (HR = 0.49, 95% CI = 0.15–1.64). As for OS, the pooled HRs for IM and TC were 0.31 (95% CI, 0.04–2.69) and 0.49 (95% CI, 0.27–0.91), respectively (Fig. 3b). The pooled HR revealed that CD8+ T cell can prolong the OS, but no statistical difference was found in the subgroup analysis of CD8+ T cells in IM (HR = 0.49, 95% CI = 0.15–1.64 *P* = 0.25; *I*^*2*^ = 81%, *P* = 0.02) (Fig. 3a). An insufficient number of studies with CD8+ T cells infiltration for CSS was acquired for meta-analysis, but single data was supplied in Table 1.

#### The prognostic value of CD45RO+T and FOXP3+ T cells on the survival of colon cancer patients

**Fig. 4:**
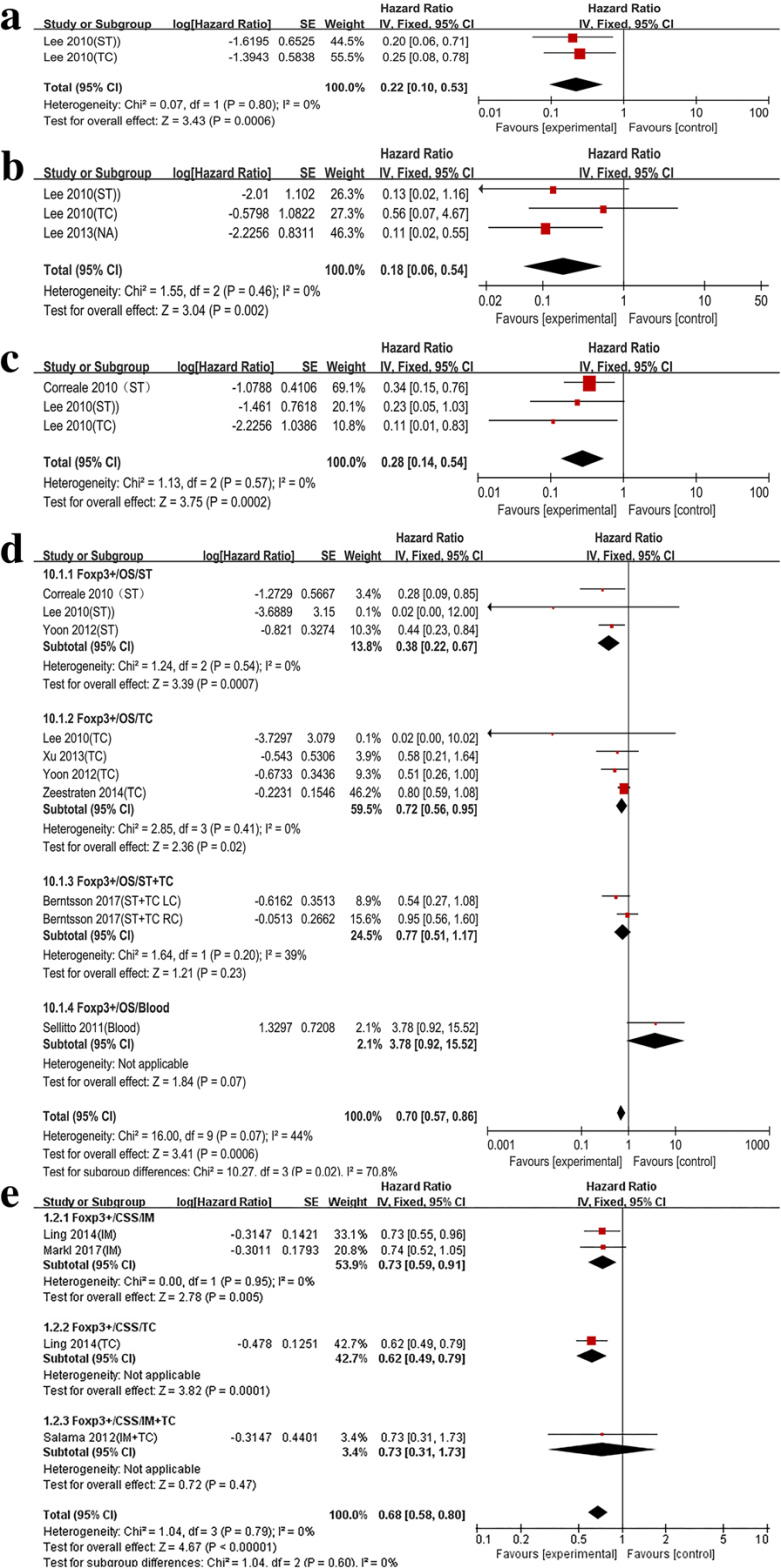
In colon cancer, forest plots of the fixed-effect subgroup meta-analysis for the efficacy of CD45RO+ T cell for DFS (**a**), OS (**b**), and Foxp3+ T cell for DFS (**c**), OS (**d**), CSS (**e**), stratified analysis based on the location of lymphocyte infiltration, including invasive tumor margin (IM), tumor center (TC), tumor stroma (ST). The horizontal bars indicate the 95% CIs. ST+TC: analysis of infiltration density of tumor infiltrating lymphocytes based on tumor stroma and tumor center; TC+IM: analysis of infiltration density of tumor infiltrating lymphocytes based on tumor stroma and invasive tumor margin; LC: left sided colon; RC: right sided colon

Two articles reported the prognostic value of CD45RO+ T cell. The pooled estimates demonstrated that CD45RO+ T cell had a positive impact on DFS (HR = 0.22, 95% CI = 0.10–0.53, *P* = 0.0006; *I*^*2*^ = 0%, *P* = 0.80) and OS (HR = 0.18, 95% CI = 0.06–0.54, *P* = 0.002; *I*^*2*^ = 0%, *P* = 0.46) (Fig. 4a, b). Among the TIL subtypes, the FOXP3+ T cell was reported by the largest number of studies. For FOXP3+ T cell, the DFS and OS displayed low and moderate heterogeneity, respectively (OS *I*^*2*^ = 0%, *P* = 0.57; DFS *I*^*2*^ = 44% *P* = 0.07). Markedly, positive pooled HRs were obtained for DFS (HR = 0.28, 95% CI = 0.14–0.54) and OS (HR = 0.70, 95% CI = 0.57–0.86) (Fig. 4c, d). In the subgroup analysis of OS, the results still revealed significant statistical differences and the heterogeneity disappeared in ST (HR = 0.38, 95% CI = 0.22–0.67, *P* = 0.0007, *I*^*2*^ = 0% *P* = 0.54) (Fig. 4d). As for CSS, in IM, the pooled results of two studies displayed significant differences (HR = 0.73, 95% CI = 0.59–0.91, *P* = 0.005; *I*^*2*^ = 0, *P* = 0.95) (Fig. 4e).

#### The prognostic value of TILs on the survival of rectal cancer patients

**Fig. 5:**
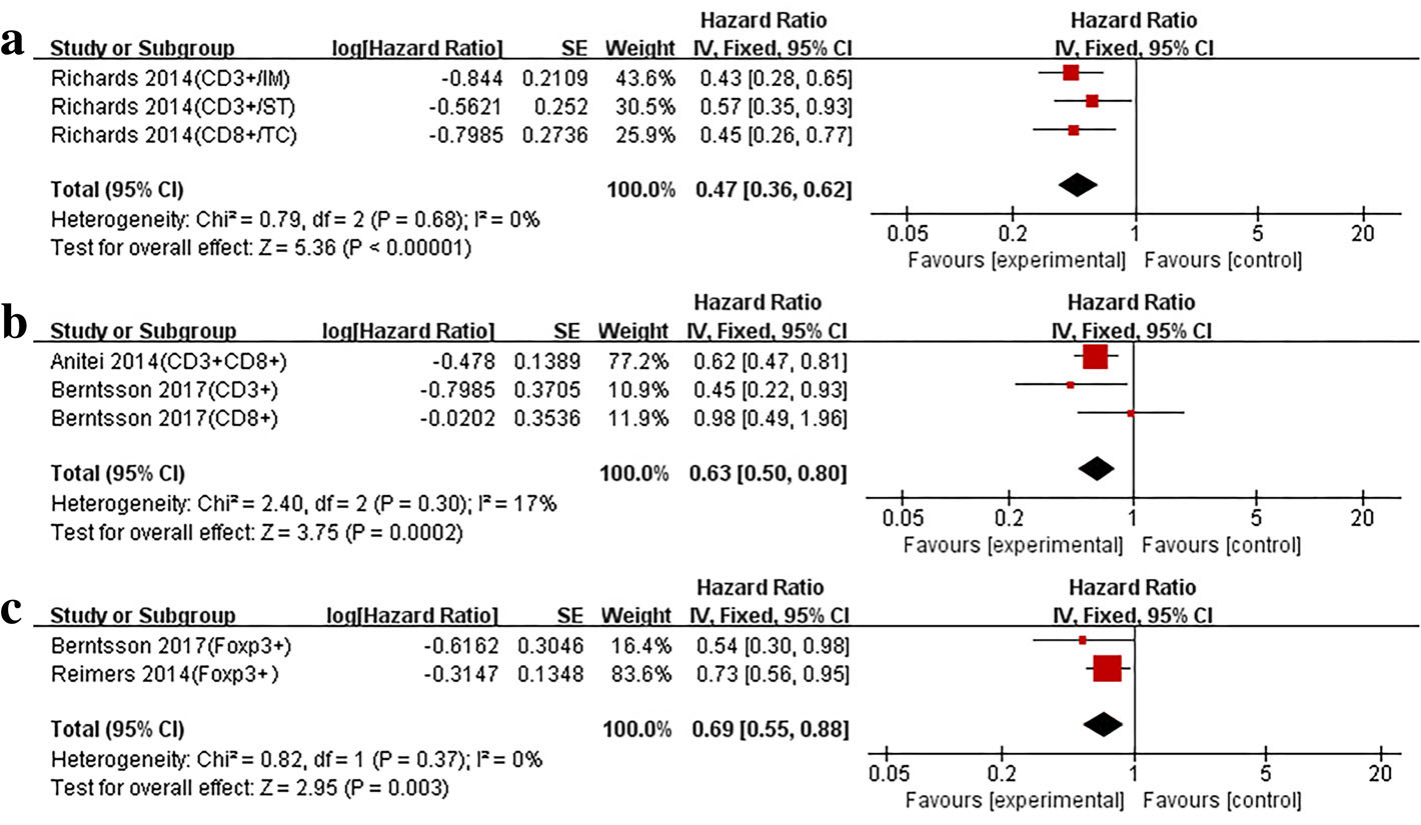
In rectal cancer, forest plots of the fixed-effect subgroup meta-analysis for the efficacy of CD3+ and CD8+T cell for CSS (**a**) and OS (**b**). The subgroup analysis of FOXP3+T cell for OS (**c**). The horizontal bars indicate the 95% CIs. CD3+/IM: analysis of infiltration density of tumor infiltrating lymphocytes based on invasive tumor margin; CD3+/ST: analysis of infiltration density of tumor infiltrating lymphocytes based on tumor stroma; CD3+/TC: analysis of infiltration density of tumor infiltrating lymphocytes based on tumor center

Only six studies were conducted for rectal cancer patients, if pooled HR was performed with single subtypes of TILs combined with different T cell counting sites, insufficient data can be acquired. Therefore, only the outcome indicators were considered (CSS and OS), and the association between two TIL subtypes and the prognosis was described. In CSS, the pooled result for CD3+ T and CD8+ T cells was 0.47 (95% CI,0.36–0.62) (*P* < 0.00001) (Fig. 5a). For OS, HR was 0.63 (95% CI, 0.50–0.80) (*P* = 0.0002) (Fig. 5b), and low heterogeneity was observed in the two groups (CSS *I*^*2*^ = 0%, *P* = 0.68; OS *I*^*2*^ = 0%, *P* = 0.30, respectively). For OS, two studies explored FOXP3+ T cell and the results were pooled (HR = 0.69, 95% CI = 0.55–0.88, *P* = 0.003) (Fig. 5c).

#### Publication bias and sensitivity analysis

**Fig. 6:**
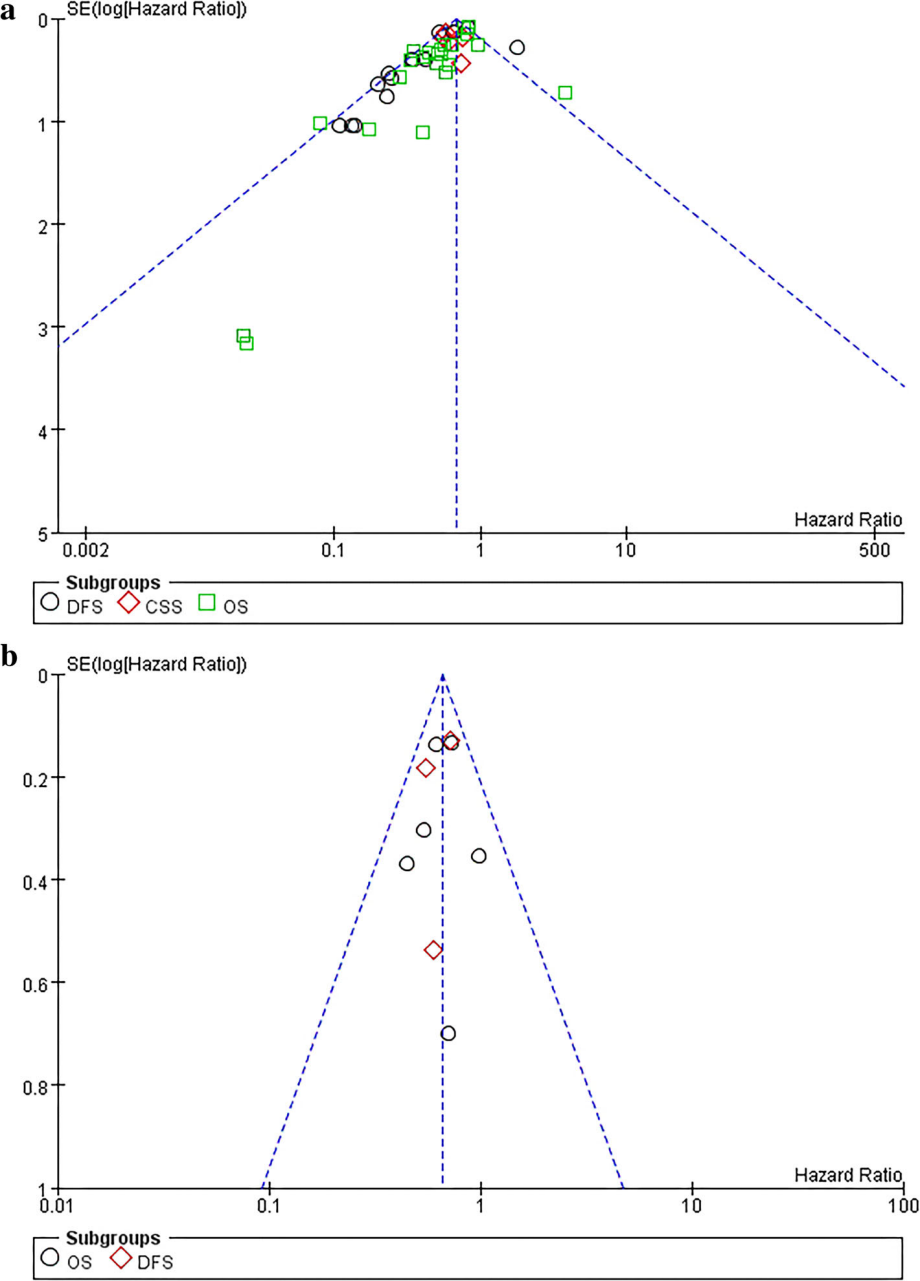
Funnel plots of the relationship between the size of the effect in individual studies and the precision of the study estimate (log (HR), horizontal axis; SE (log (HR)), vertical axis) for colon cancer (**a**) and rectal cancer (**b**). DFS disease-free survival, OS overall survival, CSS cancer-specific survival

For meta-analysis about numerous studied funnel plots indicate evidence of certain publication bias, especially for different survival indicators in the colon cancer group as a funnel plot (Fig. 6a, b). Studies were excluded one by one to observe whether the pooled results and heterogeneity were stable. The results showed that blood specimen was a major cause of heterogeneity (not presented). Moreover, partial subgroup analysis was used to assess the stability of the results and to identify sources of heterogeneity. At some counting location of TILs subset, subgroup analyses can significantly reduce heterogeneity (Figs. 2a, 3a, 4d).

## Discussion

The current meta-analysis was based on data from studies comparing high and low levels of TILs subsets in patients with colon cancer or rectal cancer. It was found that although high levels of majority of TIL subsets were associated with a favorable survival outcome (DFS, OS, or CSS), the effect of each TIL type varies when combining different survival endpoints and anatomical regions. In colon cancer, the results revealed strong evidence that CD8+, FOXP3+, and CDRO45+ T cell are correlated with increased DFS. CD3+, CD8+, and FOXP3+ T cells are correlated with improved OS, but only CD3+ T cell is associated with CSS. Subgroup analysis was performed based on the counting position of TILs, and the results from subgroup analysis were different from those of total analysis regarding DFS, and only CD3+ T cells in IM reversed the original analysis results, while CD8 + T cell in TC maintained the original statistical significance. In ST, subgroup analysis indicated that the positive prognostic value of FOXP3+ T cell on OS was not altered, and heterogeneity disappeared. From the pooled results of colon cancer, the positive prognostic value of FOXP3+ T cell in IM for OS was inconsistent from the results of a previous meta-analysis, which illustrated that high FOXP3+ T cell infiltrates were not correlated with improved OS [31]. Given that few studies performed on rectal cancer patients, the TIL subtypes were only divided into two groups for analysis, one group was CD3+, CD8+ T cells and the other was FOXP3 + T cell. The FOXP3+ T cells were analyzed separately because its prognostic effect on colorectal cancer is controversial. Overall analysis for rectal cancer showed an increased prognosis about patients with high TILs density, whether it involves CSS or OS. The sensitivity analysis demonstrated that the prognostic performance of TILs and subtypes of TILs in colon cancer or rectal cancer was stable, although the blood specimens of one study introduced heterogeneity in the meta-analysis.

The mechanisms of the prognostic effect of the location of TILs in colon cancer or rectal cancer are not known. But, numerous studies and the immune score indicated that immune cells were associated with prognosis [25, 40, 56, 57]. The CD3+ and CD8+ cells are important immune cells in colon carcinoma which determine the effect of the antitumor immune response [25, 28, 56]. Moreover, epithelial and interstitial infiltrating CD8+ T cells elicit a stronger immune response and reflect a favorable survival rate [58]. In this analysis, the prognosis of CD8+ T cells was stronger at the tumor center.

According to a previous report, the prognostic role of FOXP3+Treg in colorectal cancer is controversial. Nevertheless, in many reports, FOXP3+ TILs were positively related to the survival [21, 59]. In the analysis of different anatomical locations, it appears that FOXP3+ TILs was an independent and positive prognostic factor only in the rectum [28]. Previous studies have already discussed the reasons for the different prognostic effects of FOXP3+ TILs. It has been stated that FOXP3+ T cells are divided into two categories: FOXP3^hi^ Treg cells and FOXP3^lo^ non-Treg cells. FOXP3^hi^ Treg cells are those that highly express the transcription factor FOXP3 and are regarded as real Treg cells. Conversely, FOXP3^lo^ Treg cells do not express the naive T cell marker CD45RA or stable FOXP3 and are therefore considered to be non-inhibitory T cells. When many FOXP3^lo^ non-Treg cells infiltrated the tumor instead of FOXP3^hi^ Treg cell, they were found to have a strong prognostic value [60]. Thus, this point revealed that FOXP3+ T cells are not equivalent to Treg cells. In addition, as previously reported, only FOXP3+ Treg cell infiltration in cancer nests was associated with poor prognosis [47]. Although this meta-analysis further validated the apparently favorable prognostic value of FOXP3+ T cells in ST and not in TC, subsequent studies could consider classifying FOXP3+ T cells into functional subtypes to explore their prognostic value on different invasive sites and anatomical sites.

This meta-analysis has inevitable limitations. Although the number of studies included was sufficient, some studies did not provide detailed information regarding the subtypes of TILs or each infiltrating site. Therefore, the data used in the subgroup analysis was not sufficient which may introduce some publication bias. In addition, in the analysis of some subsets of TILs, the level of heterogeneity was relatively high, which is thanks to different cutoff values of the high-density and low-density groups of TILs, techniques of detecting TILs, and the source of specimen; one HR of study was extracted from Kaplan-Meier survival curves due to insufficient direct data of the original study, which may result in a certain data deviation. To minimize bias, we contacted the authors of one study whose data was not enough to calculate HR through email, but were not successful. In addition, the studies on rectal cancer did not conduct subgroup analysis stratified by T lymphocyte subtype due to insufficient number of studies.

Despite these limitations, the results stratified by the subtype of TILs in different location might shoulder as a positive indicator for predicting the prognosis of patients with colon cancer or rectal cancer. It is imperative to develop standard evaluation tools for TILs in colorectal cancer. In the future, more prospective studies are needed to validate on the prognostic value of TILs by dividing the colon into left and right hemi-colon with splenic flexure as the boundary. In addition, a combination of other markers such as T cell receptor (TCR), programmed death-1(PD-1), and common types of genetic mutations such as RAS mutation and BRAF mutation should be tested to design effective prognostic indicators of the so-called cold tumors which are infiltrated with low levels of TILs.

## Conclusions

In conclusion, high-density TILs or subtypes of TILs were closely associated with prolonged survival rate, especially in CC. Moreover, only high-density infiltration of some TIL subtypes at a particular infiltrating site was associated with favorable prognosis. Further prospective studies are needed to validate the prognostic value of TILs at a single counting site in colon or rectal cancer, in order to further supplement the immune score and immunotherapy targeting TILs.

## Additional files

Additional file 1: **Table S1.** General characteristics of the studies included in this meta-analysis (XLSX 15 kb)

Additional file 2: **Table S2.** The quality assessment of all included studies in this meta-analysis (XLSX 12 kb)

## Abbreviations

CI: Confidence interval
CSS: Cancer-specific survival
DFS: Disease-free survival
Foxp3+: Forkhead box P3
HR: Hazard ratio
IM: Invasive margin
K-M: Kaplan-Meier
MA: Multivariate analysis
NA: Not available
OS: Overall survival
ST: Tumor stroma
TC: Tumor center
TC+IM: The immune score including tumor center and invasion margin
TC+ST: The immune score including tumor-infiltrating lymphocytes score of tumor center and tumor stroma
TILs: Tumor-infiltrating lymphocytes
UA: Univariate analysis
